# Association between genetic liability to physical health conditions and comorbidities in individuals with severe mental illness: an analysis of two cross-sectional observational studies in the UK

**DOI:** 10.1016/S2215-0366(25)00123-3

**Published:** 2025-05-05

**Authors:** Djenifer B Kappel, Sophie E Smart, Michael J Owen, Michael C O’Donovan, Antonio F Pardiñas, James T R Walters

**Affiliations:** Centre for Neuropsychiatric Genetics and Genomics, Division of Psychological Medicine and Clinical Neurosciences, School of Medicine, Cardiff University, UK

## Abstract

**Background:**

Individuals with severe mental illness, including schizophrenia and bipolar disorder, have elevated rates of physical health conditions, contributing to increased morbidity and mortality. While environmental factors such as adverse effects from medication and lifestyle changes play a role, the contribution of genetic liability to physical health comorbidities remains underexplored. We investigated whether genetic risk for physical health conditions influences comorbidities in people with severe mental illness and compared these effects with those in the general population. Additionally, we explored the effects of psychiatric genetic liabilities and the occurrence of physical health problems in those with severe mental illness.

**Methods:**

We analysed two UK cross-sectional cohorts of people with severe mental illness—the Cardiff Cognition in Schizophrenia study (CardiffCOGS) cohort and the National Centre for Mental Health (NCMH) cohort. Individuals were selected for analyses if they responded to a validated self-report questionnaire of physical health problems and if their genetic data passed quality control. These subsets of individuals were used to test associations between polygenic risk scores for six physical health conditions (high cholesterol, type 2 diabetes, hypertension, asthma, heart disease, and rheumatoid arthritis) and corresponding physical health conditions in this population. Models were further adjusted for demographic and clinical covariates (sex, age, smoking, and clozapine use). Effect sizes from these analyses were compared in magnitude to those reported in studies conducted in the general population. We also evaluated associations between psychiatric polygenic risk scores (schizophrenia, bipolar disorder, major depressive disorder, and ADHD) and physical comorbidities. People with lived experience were involved in the analysis planning and guided the choices of outcomes analysed.

**Findings:**

Following exclusions due to missing phenotypic or genetic data (403 individuals in CardiffCOGS; 1704 individuals in NCMH), our analyses included 721 individuals from the CardiffCOGS cohort (mean age 43·7 years [SD 12·1], 267 [37·0%] females, 454 [63·0%] males, and 703 [97·5%] with self-reported White ethnicity) and 1011 from the NCMH cohort (mean age 47·6 years [SD 13·7], 553 [54·7%] females, 458 (45·3%) males, and 928 [91·8%] with self-reported White ethnicity). Polygenic risk scores for physical health conditions were associated with corresponding conditions in one or both of these cohorts, explaining between 1·4% and 6·5% of the variability in these comorbidities. Polygenic risk score effect sizes for at least one of the cohorts overlapped with the reported effects (within 84% CIs) in the general population. Adjustments for clinical and demographic factors had minimal impact on these associations. Psychiatric polygenic risk scores showed weaker and less consistent associations with physical comorbidities.

**Interpretation:**

Our findings support the role of genetic risk in physical health comorbidities among individuals with severe mental illness. Genetic liability to physical health conditions was more strongly associated with comorbidities than psychiatric genetic liability, highlighting its additive contribution alongside environmental and clinical factors. These findings indicate that there would be value in incorporating genetic risk information into predictive algorithms for physical health comorbidities in those with severe mental illness, and that polygenic risk scores should be included in research studies developing and validating such algorithms.

**Funding:**

EU Horizon 2020 programme.

## Introduction

People living with severe mental illnesses, such as schizophrenia, schizoaffective disorder, or bipolar disorder, often have comorbid physical health conditions such as cardiovascular, respiratory, metabolic, autoimmune, and inflammatory diseases.^1,2^

Moreover, they are likely to face poorer prognoses and increased mortality from these comorbidities compared with the general population.^3^ The underlying causes of the elevated burden of poor physical health in severe mental illness are not fully known. Although the prevalence of physical health comorbidities is known to increase after the onset of severe mental illness, studies have shown that this prevalence is also elevated at the point of first diagnosis of severe mental illness,^4^ implying that the comorbidities are not entirely consequences of the disorders per se, and are thus not fully explained by treatments or secondary environmental exposures such as lifestyle changes. Additional predisposing mechanisms, such as genetic factors, might affect comorbidity risk profiles among people with severe mental illness. Specifically, the increased prevalence of physical health conditions in this population could be related to genetic predisposition to physical or psychiatric disorders, or both liabilities could be jointly implicated in some way.

Research into genetic factors linked to comorbidities in people with severe mental illness has largely focused on identifying potential genetic variants, and has shown associations with both psychiatric and physical conditions.^5–8^ Genetic correlation analyses at a population level suggest the existence of loci with shared effects on psychiatric disorders and physical comorbidities; for example, genetic liability to major depressive disorder has been reported also to predispose individuals to cardiovascular disease.^9,10^ However, the overall evidence base is limited and largely inconclusive, and it is unclear whether genetic liability to any psychiatric disorder has a direct causal effect on physical health.

By contrast, studies have shown that common genetic variability associated with cardiovascular disease contributes to disease risk at least as strongly as some environmental determinants, such as smoking or physical inactivity.^11^ Although genome-wide association studies (GWAS) have begun to identify mechanisms underpinning common physical illnesses within the general population, it is unclear whether these findings generalise to those with severe mental illness, who might have quantitatively or qualitatively different exposures to relevant environmental risk factors, and studies investigating the genetic liability to physical health problems in people with severe mental illness are few. Recent studies have primarily focused on predicting antipsychotic-related adverse effects, such as the occurrence of metabolic syndromes (eg, elevated blood lipids^12^ or rapid weight gain),^13^ rather than broader physical health morbidity. These studies suggest that individuals with higher genetically inferred risk for these traits have more of these adverse effects after initiating antipsychotic treatment. However, these analyses have been mostly limited to cohorts of individuals with schizophrenia, mainly treated with antipsychotics, and their conclusions cannot be straightforwardly generalised to other severe mental illnesses, psychiatric medications, or broader physical health outcomes.

**Figure 1:**
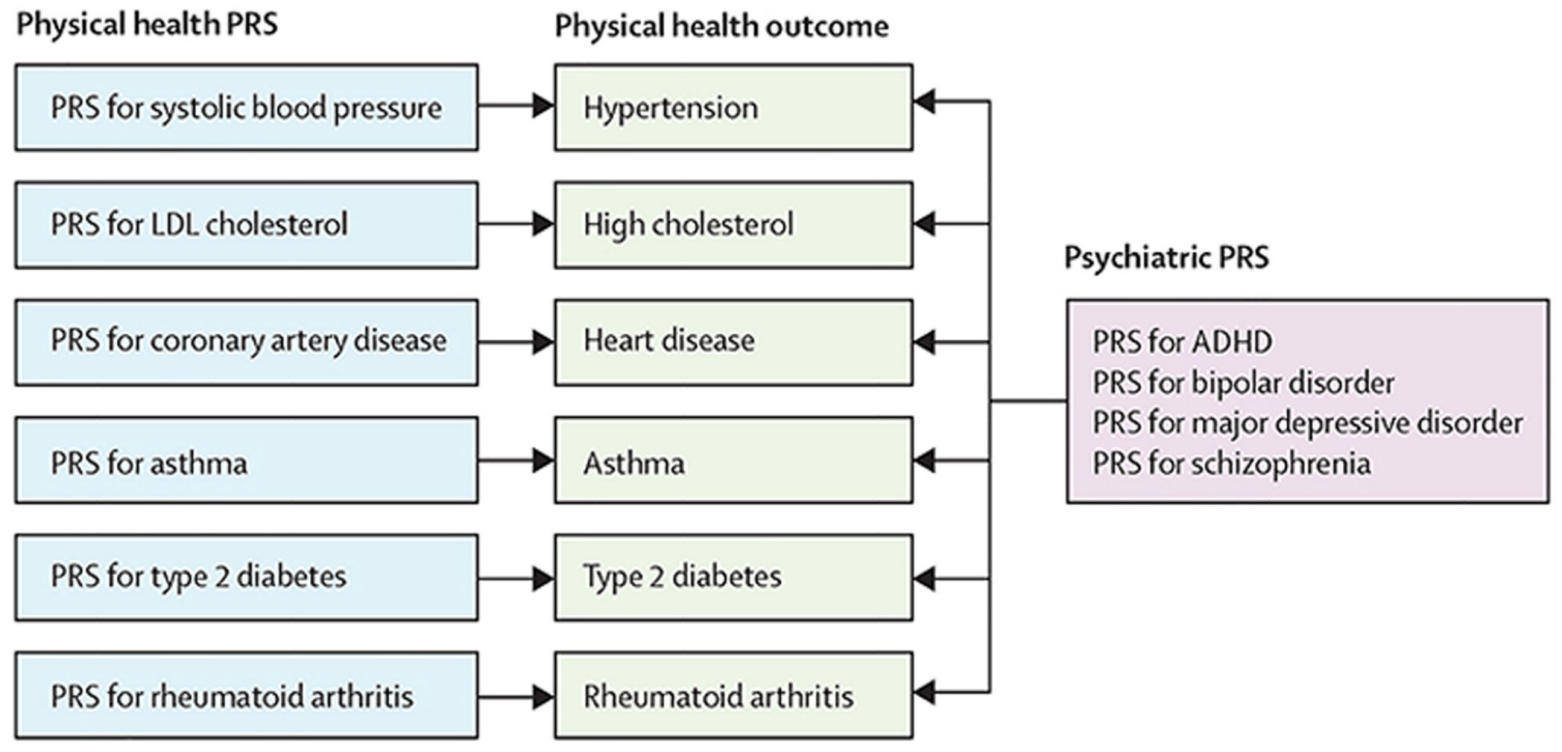
Study design The diagram illustrates the analyses performed between each PRS and physical health outcomes in severe mental illness. Arrows represent associations tested between each PRS and health outcomes. The left section shows PRSs for physical health traits and their corresponding physical health outcomes in the middle section. The right section shows PRSs for four different psychiatric conditions, with analyses being done for each PRS association with each of the individual’s physical health outcomes. PRS=polygenic risk score.

In this study, we aimed to assess whether the prevalence of a range of physical health issues in people with severe mental illness derives from genetic liability to physical health problems, and whether those effects are similar to what has been observed in the general population. Additionally, we investigated whether these genetic effects act independently of other known factors associated with physical illness comorbidity in severe mental illness. We hypothesised that polygenic risk scores for each specific physical health condition would be associated with that condition being a comorbidity in people with severe mental illness. We also explored the possibility that psychiatric polygenic risk scores might be linked to physical health outcomes (figure 1).

## Methods

### Study design and samples

We decided upon the focus of this study in consultation with people with lived experience of severe mental illness who thought physical health comorbidities should be a priority for mental health research. When designing the analytical plan, this group helped us to choose the physical health conditions that we included in this study.

The definition of severe mental illness in this study is confined to diagnoses on the psychotic spectrum. We acknowledge other diagnoses, such as severe cases of major depressive disorder, could also fall under this label. We did not include these disorders in our study as the ascertainment in our cohorts was based on either clinical diagnoses of psychotic illnesses or participant self-reports that at present have only been validated for psychotic conditions.

We analysed data from two cohorts ascertained by Cardiff University. The first, the Cardiff Cognition in Schizophrenia study (CardiffCOGS) cohort,^14^ is a clinically ascertained sample of individuals with a diagnosis of severe mental illness recruited from outpatient, in-patient, and voluntary sector mental health services in the UK (2007–17). Participants were interviewed using the Schedules for Clinical Assessment in Neuropsychiatry (SCAN),^15^ and lifetime psychiatric diagnoses were assigned based on DSM-IV or ICD-10 criteria. Participants were invited to provide a blood sample for genetic analysis. In this study, we included participants with schizophrenia, schizoaffective disorder depressed type, delusional disorder, or schizophreniform disorder (the schizophrenia spectrum group), or with bipolar disorder type 1 or 2 or schizoaffective disorder bipolar type (the bipolar spectrum group) in our severe mental illness cohort.

The second cohort, the National Centre for Mental Health (NCMH) cohort,^16^ was recruited from health-care services, voluntary organisations, or through public advertisement in Wales, UK (2012–21). A standardised assessment was administered by trained researchers; participants were asked whether a doctor or mental health professional had ever given them a mental health diagnosis from a list of potential psychiatric diagnoses.^17^ Additionally, a subset (n=105) participated in a research interview based on SCAN, and their severe mental illness status was assigned based on DSM-IV or ICD-10 criteria. Individuals reporting that their primary psychiatric diagnosis (as given by a clinical team) was schizophrenia, schizoaffective disorder depressed type, delusional disorder, or schizophreniform disorder were included in the schizophrenia spectrum group, and those with a diagnosis of bipolar disorder type 1 or 2 or schizoaffective disorder bipolar type were part of the bipolar spectrum group. A previous study using this cohort reported good validity of the self-reported severe mental illness diagnoses when assessed against a complete research interview using SCAN.^17^

NCMH was approved by the Health Research Authority and Wales Research Ethics Committee (16/WA/0323), and CardiffCOGS by the National Health Service Research Ethics Committee (07/WSE03/110). All participants provided written informed consent.

### Physical health comorbidities

For both cohorts, information on basic demographics, lifetime clinical, and environmental factors were extracted from the interviews. Additionally, each participant’s self-reported ethnicity was collected using an adapted version of the UK census categories, including the following options: Asian or Asian British; Black, African, Caribbean, or Black British; mixed or multiple ethnic groups; White (including British, Irish, Irish Traveller, Gypsy, or Roma); White from any other background; and other or unknown.

Self-reported information on physical health conditions was collected similarly across cohorts. Participants were asked to report if they had ever been told by a health-care professional that they had one or more of 20 common physical health problems. The reliability of this self-reported measure of physical health conditions has been previously validated by our group against electronic health records.^7^

From the complete list of available conditions, we analysed six physical health conditions known to have an increased prevalence in individuals with severe mental illness^4,7,18^—high cholesterol, type 2 diabetes, hypertension, heart disease, asthma, and rheumatoid arthritis. These conditions were selected due to their consistent, specific, and comparable assessment across both cohorts, and sufficient frequency (minimum 30 cases in each cohort).

### Polygenic risk scores

Participants from both cohorts provided blood or saliva for DNA analyses. Genomic data were obtained using the Illumina OmniExpress genotyping platform for CardiffCOGS and either the Illumina PsychArray or Illumina GSA platforms for NCMH. Quality control and imputation were done uniformly using the DRAGON-Data quality control pipeline (v.2.0)^19^ and the Haplotype Reference consortium, respectively. Additional information about genotyping, quality control, and imputation is provided in appendix 1 (pp 2–3).

After quality control, participants with complete phenotypic and genetic data available were included in our analyses (appendix 1 p 5).

Using the largest available GWAS summary statistics, we calculated polygenic risk scores for our physical conditions of interest (coronary artery disease, type 2 diabetes, asthma, rheumatoid arthritis, systolic blood pressure and LDL cholesterol), as well as for psychiatric conditions relevant to our definition of severe mental illness and that have been previously found to have a shared genetic component with physical comorbidities of psychiatric disorders^10^ (ADHD, bipolar disorder, major depressive disorder, and schizophrenia). The full list of GWAS summary statistics used is detailed in appendix 2 (p 3).

GWAS effect sizes and SEs were used to compute posterior single-nucleotide polymorphism (SNP) effect sizes with the polygenic score calculation method PRS-CS.^20^ Further details and parameters used for PRS-CS are in appendix 1 (pp 2–4). The PRS-CS recalculated summary statistics were then used to derive individual level polygenic risk scores for all psychiatric and physical conditions with the scoring function in PLINK 2.0. In this step, SNPs with a minor allele frequency of less than 10% were removed from the CardiffCOGS and NCMH genotypic data before polygenic risk scoring. Additionally, the extended major histocompatibility complex region (25–34 Mb) was excluded from most polygenic risk score calculations, due to its complex linkage disequilibrium structure, except for rheumatoid arthritis because of the region’s significant impact on variance explained by common genetic variants in this particular phenotype.^21^ Polygenic risk scores were scaled to a mean of zero and SD of one for subsequent association analyses.

### Statistical analysis

We conducted a within-case genetic association study to assess the relationship between polygenic risk scores for psychiatric and physical health conditions and the development of physical comorbidities in two independent samples of people with severe mental illness.

Logistic regression analyses were done using the glmrob function from robustbase in R (version 4.4), allowing for robust estimation of SEs given the low prevalence of certain physical health outcomes in these samples.

Primary minimally adjusted models were controlled for sex, age at assessment, and the top ten genomic principal components to account for population stratification in each cohort. Results are reported as effect estimate (95% CI) per SD increase in polygenic risk score. Nagelkerke’s pseudo-*R^2^* was calculated to estimate the proportion of phenotypic variance explained by each of the polygenic risk scores. As this method is not implemented for the glmrob models described above, generalised linear models with traditional SEs were fitted for this procedure.

For secondary fully adjusted models, we included additional covariates relevant to the severe mental illness population. These included the diagnosis spectrum (schizophrenia or bipolar), a history of smoking, and a history of clozapine use (as use of this antipsychotic is enriched in the CardiffCOGS sample^14^ and is associated with multiple comorbidities including weight gain and metabolic syndrome).^22^ This approach enabled us to identify whether the effects of higher comorbidity genetic risk could be confounded by these non-genetic exposures. For models with significant associations for both polygenic risk scores and clinical and environmental covariates, we did follow-up analyses to test for interaction effects between polygenic risk scores and clinical and environmental exposures.

Lastly, to examine the overall impact of the combined genetic liabilities from physical and psychiatric disorders on phenotype variability in our samples of people with severe mental illness, we extracted the residuals from the fully adjusted models for physical health polygenic risk scores described above, rank-normalised those residuals, and then analysed whether including genetic liabilities to psychiatric disorders improved any unexplained phenotype variability using linear residual regression models, including all the covariates in this model. This approach was preferred to the inclusion of additional polygenic risk score covariates in the fully adjusted models, as it can avoid failures in the model fitting process caused by sparse data.^23^

The Benjamini–Hochberg false discovery rate (FDR) method was used to correct for multiple testing at a p_FDR_≤0·1 threshold^24^ within each independent cohort.

To examine whether the results for the association of polygenic risk scores with physical health problems in individuals with severe mental illness were comparable with results from the general population, we extracted the association statistics from studies analysing the effects of physical health problem polygenic risk scores on physical health condition susceptibility in biobanks and the general population. Those studies typically do not exclude people with severe mental illness; however, given the low prevalence of severe mental illness (as defined here), the healthy volunteer bias observed in biobanks,^25^ and the association between schizophrenia genetic liability and non-participation,^26^ people with severe mental illness will represent a trivial fraction of the participants, and their inclusion could not substantially alter polygenic risk score effect size estimates. Specific details about the original studies regarding polygenic score development and phenotypes analysed are given in appendix 2 (p 4). When multiple compatible studies were found, or a study analysed more than one sample, we conducted a meta-analysis using a fixed-effects model weighted by SE, with the package metafor in R (appendix 1 pp 3–4). Comparisons of polygenic risk score effect sizes from the results in CardiffCOGS and NCMH with those extracted from studies in the general population were made by evaluating the overlap, or lack thereof, of 84% CIs, equivalent to a two-tailed two-population test with α=0·05.^27^

### Role of the funding source

The funder of the study had no role in study design, data collection, data analysis, data interpretation, or writing of the report.

## Results

We removed 130 participants from CardiffCOGS and 241 participants from the NCMH cohort for whom data were missing for more than four out of six physical health outcomes. Due to the absence of genetic data, failure of genotype quality control, or relatedness between participants (within-cohort or between-cohorts), we removed 283 participants from CardiffCOGS and 1463 participants from NCMH (appendix 1 p 2). The total number of participants excluded from each severe mental illness cohort was 403 from CardiffCOGS and 1704 from NCMH (appendix 1 p 5; appendix 2 p 1).

Among those included in the analyses, participants from the CardiffCOGS cohort (n=721) had a mean age at interview of 43·7 years (SD 12·1). 267 (37·0%) reported their sex as female and 454 (63·0%) as male. 639 (88·6%) had a schizophrenia spectrum diagnosis (schizophrenia, schizoaffective disorder depressed type, delusional disorder, or schizophreniform disorder), and 82 (11·4%) had a bipolar spectrum diagnosis (bipolar disorder type 1 or 2 or schizoaffective disorder bipolar type; table; appendix 2 p 1).

NCMH participants included in the analyses (n=1011) had a mean age of 47·6 years (SD 13·7) at the time of interview. 553 (54·7%) reported their sex as female and 458 (45·3%) as male. In this cohort, 388 (38·4%) had a schizophrenia spectrum diagnosis, and 623 (61·6%) had a bipolar spectrum diagnosis. A more complete description of characteristics of the two cohorts is given in the table and appendix 2 (p 1). The frequencies of each physical health condition by cohort and diagnostic group are presented in appendix 2 (p 2).

**Figure 2:**
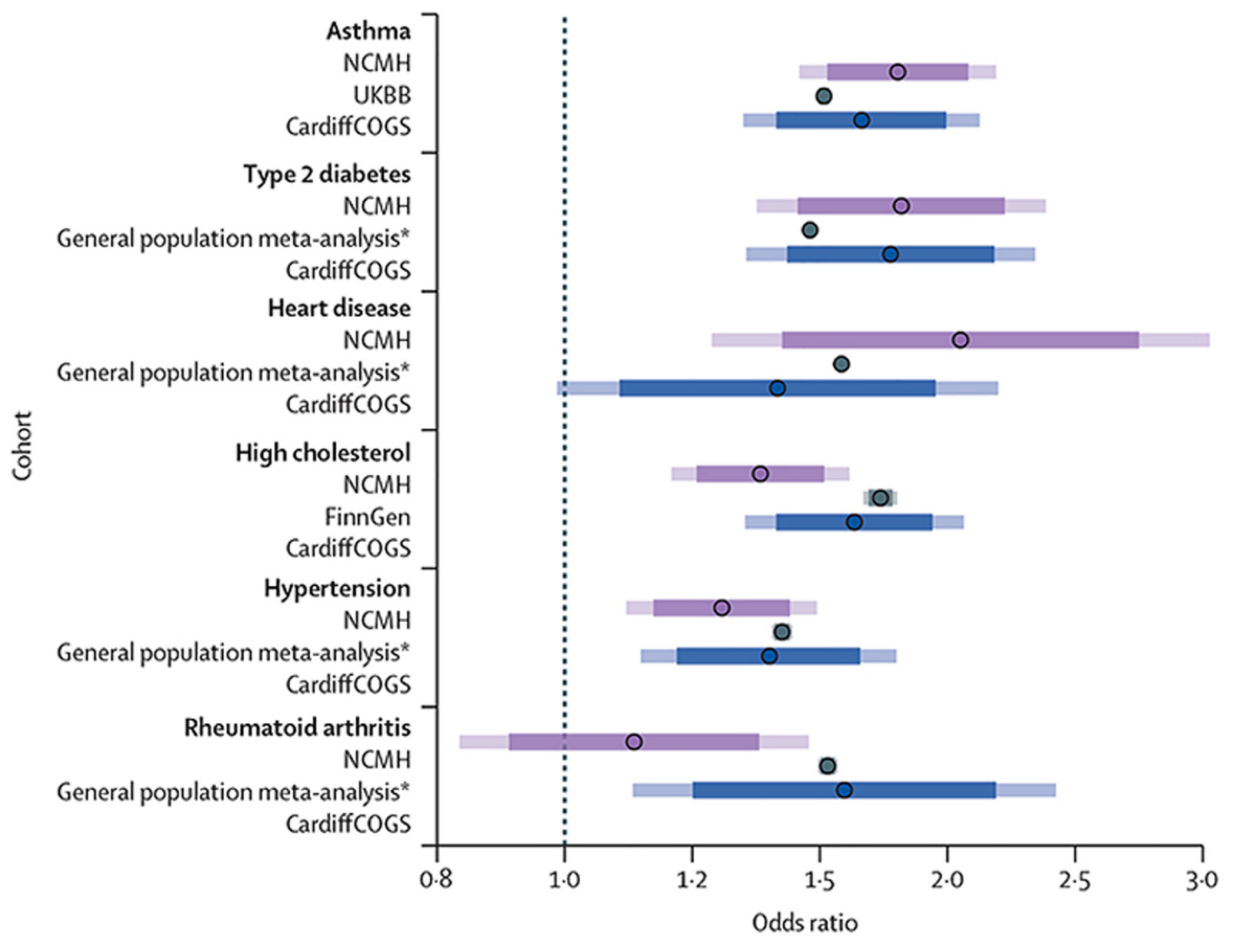
Associations between genetic liability (polygenic risk score) and physical health comorbidities, their occurrence in two cohorts of individuals with severe mental illness (NCMH [n=1011] and CardiffCOGS [n=721]), and comparison with effects observed in the general population Results are presented as odds ratios with 84% CIs (darker bars) and 95% CIs (lighter bars). Effect sizes are derived from logistic regression models adjusting for sex, age, and ten first principal components (appendix 2 pp 4–5). CardiffCOGS=Cardiff Cognition in Schizophrenia study. NCMH=National Centre for Mental Health. UKBB=UK Biobank. *See appendix 2 (p 4) for details on the meta-analysis.

All six physical health polygenic risk scores analysed were significantly associated with the presence of their corresponding physical comorbidity in one or both severe mental illness cohorts (figure 2; appendix 2 p 5). Significant polygenic risk scores explained between 1·4% and 6·5% of the observed variability of each phenotype (appendix 2 p 6). For all physical health conditions analysed, the effect sizes observed in this study (in one or both of the CardiffCOGS and NCMH cohorts) fell within the 84% CIs of the effect sizes reported in general population studies from the literature (figure 2; appendix 2 p 4).

**Figure 3:**
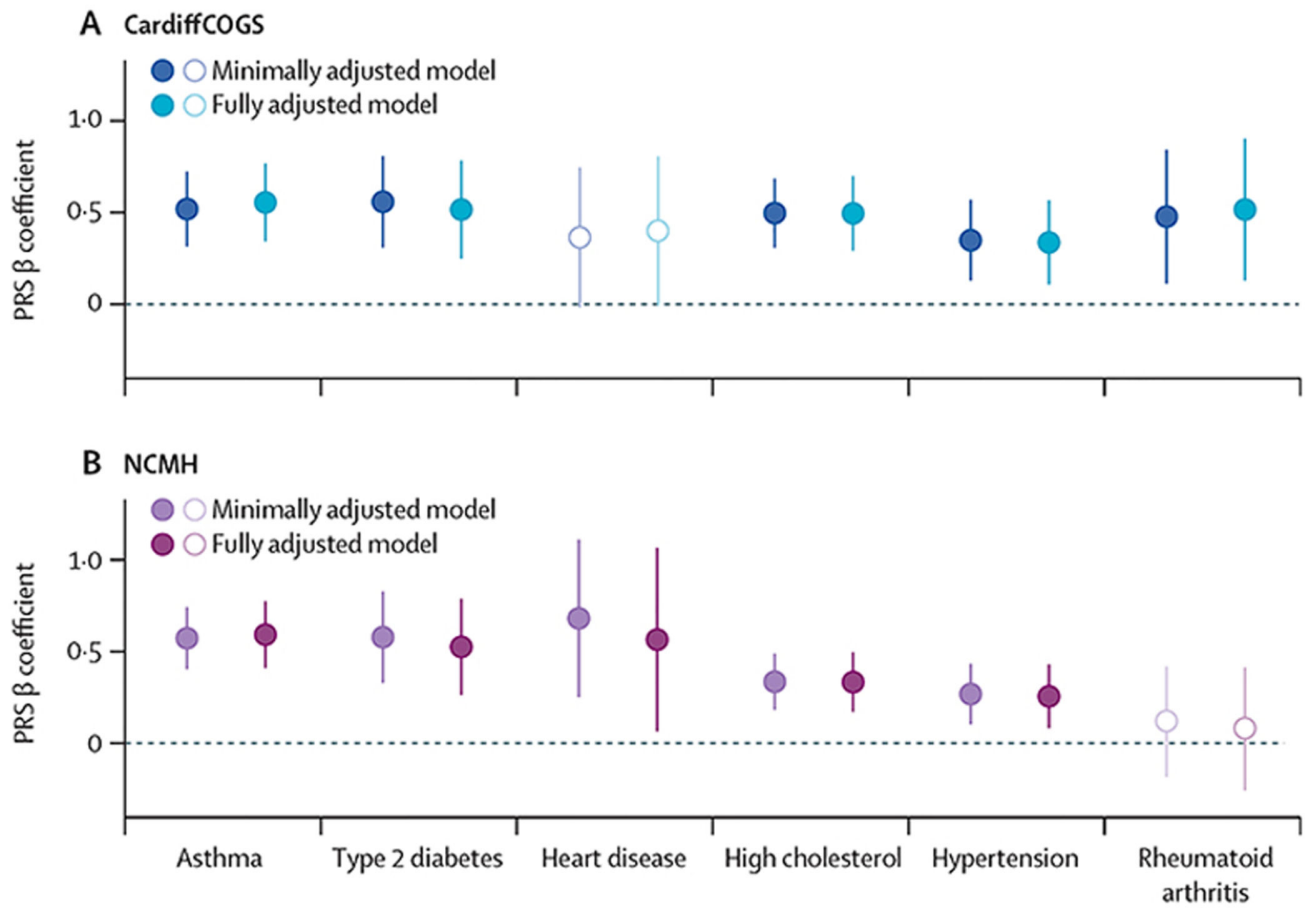
Associations between genetic liability (PRS) to physical health comorbidities and the corresponding comorbidity in two models adjusting for additional environmental covariates PRSs for physical health comorbidity are independent variables. Effect estimates are provided as β Coefficients with error bars showing 95% CIs. The results are presented for the minimally adjusted models (including covariates for sex, age, and ten first principal components), and the fully adjusted models (with the additional covariates smoking history, schizophrenia or bipolar disorder spectrum diagnosis, and use of clozapine). Solid-coloured dots indicate that the results are statistically significant at p<0·05 and false discovery rate <0·1 in at least one model in each severe mental illness cohort; unfilled dots indicate no significance at these thresholds. Details are provided in appendix 2 (p 5). CardiffCOGS=Cardiff Cognition in Schizophrenia study. NCMH=National Centre for Mental Health. PRS=polygenic risk score.

We evaluated whether the effects of polygenic risk scores were attenuated in models adjusting for well established clinical, demographic, and environmental risk factors of physical conditions (sex, age, smoking, and clozapine use; appendix 2 p 5). In these fully adjusted models, we observed minimal changes to the regression estimates and the associations between polygenic risk scores and their comorbidities remained statistically significant (FDR<0·1) in at least one cohort, except for the association of polygenic risk score for coronary artery disease with heart disease (figure 3; appendix 2 p 5). Heart disease had the lowest prevalence among the comorbidities analysed, with 35 individuals in the CardiffCOGS cohort and 42 individuals in the NCMH cohort reporting heart problems.

Of the identified non-genetic risk factors, age and sex showed consistent associations across physical health outcomes, and clozapine use was associated with higher LDL cholesterol and type 2 diabetes (appendix 2 p 5). However, we did not detect an interaction effect of physical illness polygenic risk scores and clozapine use when analysing these comorbidities (appendix 2 p 7).

**Figure 4:**
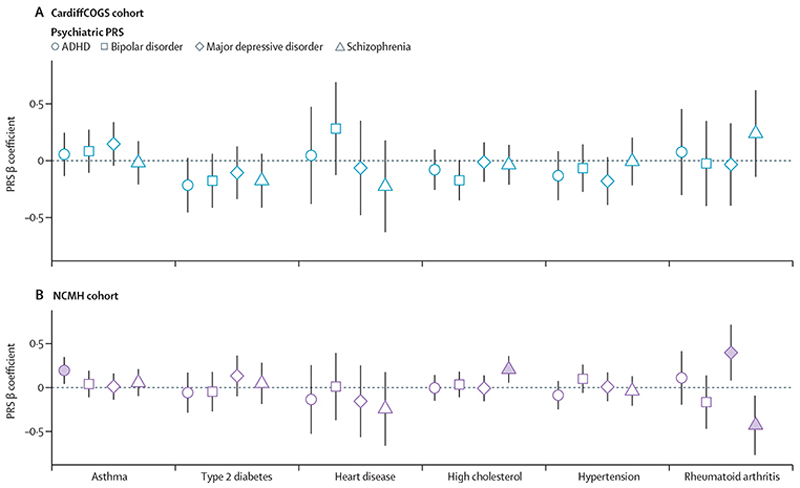
Associations between physical health comorbidities and four psychiatric PRSs in two cohorts of individuals with severe mental illness (NCMH [n=1011] and CardiffCOGS [n=721]), after correction for sex, age, and ten principal components The symbols indicate the estimated regression effect size (β coefficient) for each analysed PRS and comorbidity outcome, with error bars indicating 95% CIs. Additional details are provided in appendix 2 (pp 8–9). Solid-coloured shapes indicate that a significant association was observed (false discovery rate <0·1); unfilled shapes indicate no significance. CardiffCOGS=Cardiff Cognition in Schizophrenia study. NCMH=National Centre for Mental Health. PRS=polygenic risk score.

We next tested whether genetic liability to four psychiatric disorders (ADHD, bipolar disorder, major depressive disorder, and schizophrenia) was associated with physical health comorbidity in people with severe mental illness. Although we identified some associations (FDR<0·1; figure 4, appendix 2 p 8), the overall evidence for an effect of psychiatric polygenic risk scores on comorbidity outcomes was inconsistent. We observed significant differences in effect estimates between the CardiffCOGS and NCMH cohorts for the associations of polygenic risk scores for schizophrenia with both high cholesterol and rheumatoid arthritis, suggesting heterogeneity in these associations across cohorts.

Given the strong association between physical health traits and their corresponding comorbidities identified above, we also analysed whether psychiatric polygenic risk scores could help to explain additional phenotypic variability in the occurrence of physical problems in people with severe mental illness (appendix 2 p 9). We observed an association of polygenic risk scores for schizophrenia with high cholesterol while controlling for other genomic, clinical, and environmental factors in the NCMH cohort. As reported for the initial association between psychiatric disorder polygenic risk scores and physical comorbidity, this finding was not consistent between the two severe mental illness cohorts.

## Discussion

The elevated rates of these comorbidities in the severe mental illness population are often linked to demographic and lifestyle characteristics in those with severe mental illness, such as poor diet, sedentary behaviour, smoking, and side-effects of pharmacological treatment with antipsychotics, mood stabilisers, and antidepressants.^28^ In this study, we evaluated whether—in addition to the known clinical and environmental factors associated with the development of comorbidities in people with severe mental illness—biological mechanisms such as genetic risk associated with psychiatric and physical illnesses could affect the development of physical health problems in this population. We observed that the occurrence of high cholesterol, type 2 diabetes, hypertension, asthma, heart disease and rheumatoid arthritis in people with severe mental illness were statistically significantly associated with genetic liability to these conditions.

Given previous results suggesting that phenotypic correlations between severe mental illness and physical comorbidities seems to be largely driven by environmental factors,^10^ we also examined whether the effects of polygenic risk scores for physical health problems and the occurrence of these outcomes in our samples could be modified by demographic, clinical, and environmental factors known to increase the risk for comorbidity in those with severe mental illness. Our inclusion of covariates, such as smoking and medication use, did not significantly alter the observed associations between polygenic risk scores for physical health conditions and the corresponding clinical outcomes. Moreover, by comparing the effect size estimates for physical health polygenic risk scores and comorbidities obtained in our two severe mental illness cohorts with estimates from studies done in biobanks, we found that genetic variants associated with physical disorders conferred similar risks of comorbidities in individuals with severe mental illness and in the general population. These findings suggest that, despite the probable roles of psychotropic medication use and behavioural and lifestyle characteristics in the development of some physical health comorbidities in individuals with severe mental illness, genetic liability to physical health conditions influences these conditions in people with severe mental illness to an extent similar to that in the rest of the population.

Several studies have reported correlations between the genetic liability to psychiatric disorders and genetic liability to physical health conditions using studies conducted in the general population, suggesting a link between psychiatric genetic risk and the occurrence of physical health conditions. In this study, we found limited evidence for the association of psychiatric genetic liability (indexed by polygenic risk scores) and the occurrence of physical health comorbidities in individuals with severe mental illness. The effects of psychiatric polygenic risk scores on comorbid physical conditions observed were weaker and more variable across the cohorts than physical health polygenic risk scores and, in some cases, did not significantly contribute to the explained variance of the comorbid physical condition. Despite the previously reported genetic overlap,^5,8,9^ our findings suggest that the occurrence of physical health comorbidities among people with severe mental illness is not substantially influenced by common genetic variability associated with psychiatric disorders. Instead, other severe mental illness-related factors—eg, the effects of medication, illness severity, socioeconomic adversity, and other behavioural and psychosocial factors, such as loneliness^29^ or smoking^30^— might affect the development of comorbidities.

Recognising the high prevalence of comorbid physical health conditions in individuals with severe mental illness, there is a drive for services to integrate physical health monitoring, including metabolic and cardio-vascular risk assessments, into their clinical management plans for people with severe mental illness.^1,31^ Although risk assessment tools for physical health conditions are widely used in the general population, and there is increasing support for the inclusion of genetic risk factors in those tools,^11,32,33^ it is unclear whether such tools are equally applicable to individuals with severe mental illness.^34^ Recent longitudinal studies have suggested that individuals with severe mental illness carrying higher genetic risk for physical or metabolic conditions were more likely to have cholesterol level and BMI increases during pharmacological treatment with antipsychotics.^12,13^ The validation and integration of polygenic risk scores into clinical practice could enable more personalised interventions, where genetic risk scores serve as complementary tools for early screening and treatment optimisation in individuals predisposed to physical health problems.^35^ Our findings provide initial evidence that, if genetic information is integrated into stratification and prediction algorithms in the general population, it could be equally valuable for individuals with severe mental illness. Because the effects of genetic liability to physical health problems in people with severe mental illness are indistinguishable from the effects observed in the general population, strategies to address comorbid health problems in individuals with severe mental illness might benefit from the adoption of the same health-promoting habits (eg, well balanced diets, smoking cessation, and physical exercise) and adherence to relevant medical interventions (eg, use of antihypertensives or statins) that are effective in the general population.

While our study provides robust evidence for the role of genetic risk in physical comorbidities among individuals with severe mental illness, several limitations should be acknowledged. First, our design does not allow us to directly examine whether individuals with severe mental illness have significantly higher genetic liability to comorbidities than individuals without psychiatric disorders recruited from the same population, or whether this would explain part of the elevated prevalence of some physical health problems among people with severe mental illness.

Second, despite our efforts to control for confounding factors, we were unable to account for many important variables—such as BMI, weight changes, poor diet, sedentary behaviour, and socioeconomic status—which are known to affect the occurrence of physical comorbidities. Additionally, the small sample size of some comorbidities we studied, such as heart disease, might have reduced statistical power, particularly when adjusting for covariates.

Third, while we tried to avoid excluding participants based on ancestry, most individuals in our sample were of European descent, and at least one of our analyses had to be restricted to this subpopulation. We used multi-ancestry GWAS summary statistics, if available, but the generalisability of our findings to non-European populations remains uncertain. Future studies should replicate these findings in more diverse cohorts to ensure broader applicability.

Finally, while this study focuses on a specific subset of physical health conditions that are prevalent and clinically relevant, other physical health conditions not included in our analysis could have different genetic or environmental factors that influence physical health comorbidities in those with severe mental illness. Further research is needed to explore a wider range of physical health conditions in populations with severe mental illness, particularly those that are understudied yet might be relevant to this population’s overall health and quality of life.

In summary, this study provides strong evidence supporting the role of genetic risk in the development of common physical health conditions in individuals with severe mental illness. Our findings indicate that the occurrence of these comorbidities is significantly more affected by the genetic liability to physical health conditions than by psychiatric genetic liability. These genetic effects appear to act independently of established environmental risk factors in contributing to comorbidity development. Further research is required to assess whether these findings can inform stratification and prediction models, with the potential to enhance the development of personalised medicine approaches in psychiatric care.

## Figures and Tables

**Table:** Characteristics of participants included in the analyses

	CardiffCOGS cohort (n=721)	NCMH cohort (n=1011)
Mean age at assessment, years	43·7 (12·1)	47·6 (13·7)
Sex		
Male	454 (63·0%)	458 (45·3%)
Female	267 (37·0%)	553 (54·7%)
Missing data	0	0
Ever been married or cohabited		
No	493 (68·4%)	286 (28·3%)
Yes	220 (30·5%)	712 (70·4%)
Missing data	8 (1·1%)	13 (1·3%)
Have children		
No	262 (36·3%)	464 (45·9%)
Yes	177 (24·5%)	539 (53·3%)
Missing data	282 (39·1%)	8 (0·8%)
Educational attainment		
A-levels*	162 (22·5%)	249 (24·6%)
Apprenticeship or other	24 (3·3%)	31 (3·1%)
Undergraduate or postgraduate degree	107 (14·8%)	258 (25·5%)
GCSEs†	263 (36·5%)	297 (29·4%)
No qualification	146 (20·2%)	159 (15·7%)
Missing data	19 (2·6%)	17 (1·-7%)
Current employment		
Employed	71 (9·8%)	188 (18·6%)
Homemaker	0	11 (1·1%)
Other	15 (2·1%)	44 (4·4%)
Retired	25 (3·5%)	65 (6·4%)
Unable to work due to illness	456 (63·2%)	508 (50·2%)
Unemployed	117 (16·2%)	53 (5·2%)
Voluntary	23 (3·2%)	131 (13·0%)
Missing data	14 (1·9%)	11 (1·1%)
Ever been a regular smoker		
No	168 (23·3%)	318 (31·5%)
Yes	546 (75·7%)	687 (68·0%)
Missing data	7 (1·0%)	6 (0·6%)
SMI diagnosis group		
Schizophrenia spectrum‡	639 (88·6%)	388 (38·4%)
Bipolar spectrum§	82 (11·4%)	623 (61·6%)
Missing data	0	0
Ever taken clozapine		
No	434 (60·2%)	815 (80·6%)
Yes	273 (37·9%)	189 (18·7%)
Missing data	14 (1·9%)	7 (0·7%)
Ethnicity (self-reported)		
Asian or Asian British	4 (0·6%)	23 (2·3%)
Black, African, Caribbean, or Black British	3 (0·4%)	16 (1·6%)
Mixed or multiple ethnic groups	10 (1·4%)	22 (2·2%)
White (British, Irish, Irish Traveller, Gypsy or Roma)	691 (95·8%)	901 (89·1%)
White from other background	12 (1·7%)	27 (2·7%)
Other or unknown	1 (0·1%)	22 (2·2%)
Missing data	0	0

CardiffCOGS=Cardiff Cognition in Schizophrenia study. GCSE=General Certificate of Secondary Education. NCMH=National Centre for Mental Health. SMI=severe mental illness.

* Academic qualifications in the UK typically taken by students aged 14–16 years at the end of secondary school (years 10 and 11), equivalent to the US High School Diploma.

† Academic qualifications in the UK typically taken by students aged 16–18 years after completing GCSEs, equivalent to Advanced Placement in the USA or the International Baccalaureate Diploma.

‡ Includes schizophrenia, schizoaffective disorder depressed type, delusional disorder, and schizophreniform disorder.

§ Includes bipolar disorder type 1 or 2 and schizoaffective disorder bipolar type.

## Data Availability

To comply with the ethical and regulatory framework of both CardiffCOGS and NCMH studies, access to individual-level data requires a collaboration agreement with Cardiff University for CardiffCOGS, and with the NCMH Core Team for NCMH data. Requests to access de-identified datasets, data dictionaries, and other summaries should be directed to the corresponding author.

## Research in context

### Evidence before this study

We conducted a systematic search of literature to examine the role of genetic liability in physical health comorbidities among individuals with severe mental illness. We searched PubMed and Cochrane for articles in English, from Jan 1, 2000 to Jan 31, 2025, using the following terms: ((“severe mental illness” OR “SMI” OR “schizophrenia” OR “bipolar disorder”) AND (“polygenic risk score” OR “PRS” OR “genetic risk” OR “polygenic score”) AND (“physical health” OR “comorbidity” OR “multimorbidity”)). Previous research has established that individuals with severe mental illness have elevated rates of physical health conditions, and that these are associated with environmental factors such as medication adverse effects and lifestyle changes. However, the role of genetic liability to physical health conditions in this population remains underexplored. Few studies have investigated whether polygenic risk scores for physical health conditions are associated with comorbidities in severe mental illness, and none have systematically compared these effects with those observed in the general population. Our study aimed to address these gaps by evaluating the contribution of genetic risk to physical health comorbidities in individuals with severe mental illness and comparing these effects to those in the general population.

### Added value of this study

To our knowledge, this is the first study to comprehensively assess the role of genetic liability in physical health comorbidities among individuals with severe mental illness using polygenic risk scores for six common physical health conditions (high cholesterol, type 2 diabetes, hypertension, asthma, heart disease, and rheumatoid arthritis). We used data from two well characterised cohorts of individuals with severe mental illness in the UK (the Cardiff Cognition in Schizophrenia study cohort and the National Centre for Mental Health cohort). Our findings show that polygenic risk scores for physical health conditions are consistently associated with the presence of corresponding conditions in individuals with severe mental illness, with effect sizes similar to those reported in the general population. Importantly, these associations remained significant after adjusting for environmental and clinical factors, highlighting the additive contribution of genetic risk to physical health comorbidities in this population. By contrast, psychiatric polygenic risk scores showed weaker and less consistent associations with physical comorbidities, underscoring the specificity of genetic liability to physical health conditions in driving multimorbidity in people with severe mental illness. These findings indicate that the genetic component of physical health risk operates through mechanisms that are partly independent of the causes and clinical context of severe mental illness.

### Implications of all the available evidence

Our findings have important implications for understanding and addressing physical health comorbidities in individuals with severe mental illness. The strong association between genetic liability to physical health conditions and comorbidities suggests that genetic risk contributes in addition to environmental and clinical factors in driving multimorbidity in this population, and that these effects appear to be no different from what is observed in the general population. Therefore, integrating genetic information into stratification and prediction algorithms for physical health comorbidities could be equally valuable for individuals with severe mental illness. This approach could help to identify individuals at elevated risk due to exposures to known environmental risk factors—such as medication use and smoking—and could improve the management, prevention, and monitoring of physical health comorbidities in this population.
